# Social, economic, and environmental disparities in device-measured 24-hour movement behaviors in a nationally representative cohort of older English adults

**DOI:** 10.1093/gerona/glag197

**Published:** 2026-08-19

**Authors:** Laura Brocklebank, Andrew Steptoe, Mikaela Bloomberg, Aiden Doherty

**Affiliations:** Big Data Institute, Nuffield Department of Population Health, University of Oxford, Oxford, United Kingdom; Department of Behavioural Science and Health, University College London, London, United Kingdom; Department of Epidemiology and Public Health, University College London, London, United Kingdom; Big Data Institute, Nuffield Department of Population Health, University of Oxford, Oxford, United Kingdom; (Medical Sciences Section)

**Keywords:** Physical activity, Sedentary behavior, Sleep, Accelerometry, Socioeconomic disparities

## Abstract

**Background:**

Insufficient physical activity, excessive sedentary time, and suboptimal sleep duration (ie, not excessively short or long) are linked to morbidity and premature mortality and may contribute to health inequalities, but device-measured, nationally representative data capturing the full 24-hour movement spectrum remain scarce, particularly among older adults. This study examined social, economic, and environmental disparities in 24-hour movement behaviors in the 2021-2023 English Longitudinal Study of Aging (ELSA) accelerometry substudy.

**Methods:**

A subset of 5382 ELSA participants (71.9%) was invited to wear a wrist accelerometer for eight days, with 4354 (80.9%) agreeing. Raw data were processed using three distinct machine learning algorithms to estimate step count, sleep duration, moderate-to-vigorous and light physical activity, and sedentary time. Cross-sectional associations with sex, age, education, marital status, wealth, and urbanicity were examined using linear regression.

**Results:**

Data from 3648 participants (mean age 68.5 ± 9.3 years; 44.3% male) were included in the wear time analyses (median 6.6 days, IQR 6.0-6.9), and data from 3161 (86.7%) participants with sufficient wear time were included in the 24-hour movement behavior analyses. Older, unmarried, or lower education/wealth participants were less active, more sedentary, and slept less. Rural participants were more active than urban participants. Females accumulated fewer steps, less moderate-to-vigorous activity, and more sedentary time, but more light activity and longer sleep than males.

**Conclusions:**

Social, economic, and environmental disparities exist across the full 24-hour movement spectrum, highlighting groups for targeted interventions. Follow-up will clarify how 24-hour movement behaviors influence healthy aging and contribute to health inequalities.

## Introduction

The full 24-hour movement spectrum consists of physical activity, sedentary behavior, and sleep. Insufficient physical activity, excessive sedentary time, and suboptimal sleep duration (ie, not excessively short or excessively long) are all independently associated with premature mortality and a higher risk of many chronic diseases.^1^^,^^2^ These 24-hour movement behaviors may also reflect underlying social determinants of health and contribute to social inequalities in health.^3^^,^^4^

Since 2004, the World Health Organization (WHO) has advocated for the systematic monitoring of physical activity in national surveillance systems. However, monitoring has largely focused on moderate-to-vigorous physical activity rather than the full 24-hour movement behavior spectrum. Moreover, the most widely used method to date—questionnaires—has several limitations that can lead to highly variable estimates of guideline adherence. These include recall and social desirability biases, inconsistencies in how behaviors are measured, and cultural differences that hinder cross-country comparisons.

Accelerometers can provide more accurate and comparable estimates of 24-hour movement behaviors. However, there is currently a lack of device-measured data from nationally representative prospective cohort studies,^5^ particularly among older adults, whose behaviors may differ substantially from those of younger and middle-aged adults.^6^ Of the few studies with device data, the most common device placement has been the waist. Waist-worn devices are typically worn only during waking hours, which reduces overall wear time and prevents the capture of sleep and therefore the full 24-hour movement behavior spectrum. In addition, these studies have mainly relied on proprietary metrics rather than raw acceleration data, limiting transparency and reducing comparability and consistency across studies and over time.

Raw acceleration data from wrist-worn accelerometers, worn continuously for 7-9 days, have been collected in several large-scale international epidemiological cohorts to measure 24-hour movement behaviors, including the UK Biobank (UKB),^7^ the British Whitehall II study,^8^ the 2011-2014 US National Health and Nutrition Examination Survey (NHANES),^9^ the FinHealth 2017 study,^10^ the Observation of Cardiovascular Risks and Health in Luxembourg study (ORISCAV-LUX 2),^11^ the Rotterdam study,^12^ the China Kadoorie Biobank (CKB),^13^ and the Pelotas birth cohorts.^14^ However, only three of these are nationally representative.^9–11^ None of these nationally representative studies focused specifically on older adults or examined movement behaviors spanning the full 24-hour spectrum. Moreover, while all three studies examined disparities by age, sex, and education, only one considered broader social and economic factors such as marital status and wealth,^15^ and none examined urbanicity. These characteristics may influence 24-hour movement behaviors through pathways including social support,^3^ work strain and stress,^3^^,^^11^^,^^15^ financial resources,^11^ and access to natural environments that encourage physical activity,^3^^,^^16^ leaving important gaps in our understanding of how 24-hour movement behaviors reflect underlying social determinants of health and contribute to social inequalities in health.

Therefore, the primary aim of this study was to examine social, economic, and environmental disparities in 24-hour movement behaviors in the 2021-2023 accelerometry substudy of the nationally representative English Longitudinal Study of Aging (ELSA). A secondary aim was to explore daily and diurnal patterns in 24-hour movement behaviors in this population.

## Materials and methods

### Study population

ELSA is an ongoing nationally representative prospective cohort study of the English population aged ≥50 years. Data collection began in 2002-2003 with follow-up every two years. Details of survey design are available elsewhere.^17^ Wave 10 of ELSA received ethical approval from the South Central-Berkshire Research Ethics Committee on March 22, 2021 (21/SC/0030). All participants gave written informed consent, and all procedures were conducted in accordance with the principles of the Declaration of Helsinki.

Between June 2021 and October 2022 (Wave 10), approximately 75% of households (including core members and partners) interviewed for the main study were randomly selected and invited to additionally wear an activity monitor for eight consecutive days and nights to capture their usual levels of physical activity. Subsequently, to mitigate low response due to COVID-19, all households interviewed for the main study between November 2022 and March 2023 were invited to participate in the accelerometry substudy.

### Data collection

The Axivity AX3 (Axivity Ltd., Newcastle, UK) is a wrist-worn triaxial accelerometer that has been used to measure 24-hour movement behaviors in other large-scale epidemiological cohorts, such as UKB^7^ and CKB.^13^ The accelerometers were set to start recording at 10 am two working days after postal dispatch and stop recording ten full days later, including three additional days to allow for postal delays. The accelerometers were set to capture triaxial acceleration data at 100 Hz with a dynamic range of ± 8 g.

Participants were asked to start wearing the accelerometer immediately after receiving it in the post and wear it on their dominant wrist 24 hours per day for eight consecutive days. Participants were also asked to record on an activity monitoring postcard the date and time when they started wearing the accelerometer. After eight days, participants were asked to mail both the accelerometer and postcard back to the coordinating center in a pre-paid envelope. In cases where the accelerometer was not returned, the coordinating center contacted the participant up to four times (by text message, email, or phone call) to confirm its status. Participants were informed that they could wear the waterproof accelerometer when bathing or swimming but not in extremely high temperature or pressure environments (eg, in a sauna or when diving). Participants were asked to carry on with their normal activities while wearing the accelerometer and did not receive feedback on their activity levels until after it was returned.

Information on participants’ social, economic, and environmental factors was collected through computer-assisted telephone, video, or in-person interviews according to prevailing COVID-19 restrictions. Social factors included sex (male or female), age (50-59, 60-69, 70-79, or ≥80 years), and marital status (married or unmarried); economic factors included education (high, intermediate, or low) and household non-housing wealth (quintiles); and environmental factors included urbanicity (urban or rural). Further details on the derivation and classification of these variables can be found in Table S1.

### Data processing

Rather than processing the full ten-day monitoring periods, which could have included time when the accelerometer was in transit, we used data from the activity monitoring postcards to define participant-specific seven-day monitoring periods. The start time was set to 00:00 on the day after the participant reported putting the accelerometer on, or 00:00 on the second day of the ten-day monitoring period if postcard data were unavailable. The end time was seven full days after the start time.

The accelerometer-based outcomes considered in the present analysis were total wear time (days), overall daily step count (steps/d), overnight sleep duration (h/night), time spent in moderate-to-vigorous physical activity (min/d), time spent in light physical activity (h/d), and total sedentary time (h/d). Three different machine learning algorithms were applied to the raw acceleration data to derive estimates of these outcomes. Exclusion criteria, including the definition of non-wear time, the definition of a valid day, and the number of valid days required, differed slightly across the three algorithms and are therefore reported separately for each. To be included in the final analysis, participants were required to have valid data for all accelerometer-based outcomes across all three algorithms.

#### Overall daily step count

Overall daily step count (steps/d) was derived using a hybrid machine learning and peak detection algorithm (https://github.com/OxWearables/stepcount, v3.8.0) and was reported as the median number of steps per day across the seven-day monitoring period. This algorithm was evaluated in 69 adults under both free-living (*n* = 39) and laboratory (*n* = 30) conditions using either a foot-facing or third-person video camera as the reference measure.^18^ Participants were excluded if the data could not be parsed, the device could not be calibrated, more than 1% of readings were ‘clipped’ (ie, fell outside ±2 g) before or after calibration, or if average acceleration was implausibly high (>100 mg). Participants were also excluded if they did not have sufficient wear time (≥3 days of data with coverage across every one-hour period of the 24-hour cycle). Non-wear time was defined as unbroken episodes of ≥90 minutes in which the standard deviation (*SD*) of acceleration on each axis was <13 mg. To account for potential diurnal bias in wear time, recording interruptions and non-wear periods were imputed using the average value from the corresponding minute across the remaining valid days.

#### Overnight sleep duration

Overnight sleep duration (h/night) was derived using a sleep staging algorithm (https://github.com/OxWearables/asleep, v0.4.13). The *asleep* algorithm classifies each 30-second epoch of acceleration data into one of the three sleep stages: (1) wake, (2) rapid eye movement sleep (REM), and (3) non-rapid eye movement sleep (NREM). Overnight sleep duration was defined as the length of the longest sleep window within each noon-to-noon interval and was reported as the average across the seven-day monitoring period. The longest sleep window could include brief wake periods of up to 60 minutes without being split into separate windows. This 60-minute threshold was applied to each wake period and is a predefined parameter within the algorithm, reflecting a pragmatic, human-informed approach to distinguishing brief awakenings (eg, turning over in bed, using the bathroom, or checking the time) from sustained wakefulness. This algorithm was evaluated in 1166 adults using laboratory-based polysomnography (PSG) as the reference measure.^19^

Participants were excluded if the data could not be parsed, the device could not be calibrated, more than 1% of readings were ‘clipped’ (ie, fell outside ±3 g) before or after calibration, or if average acceleration was implausibly high (> 200 mg). Participants were also excluded if they did not have sufficient wear time (≥ 22 h/d for ≥3 days, including ≥1 weekend day). Non-wear time was defined as unbroken episodes of ≥90 minutes in which the *SD* of acceleration on each axis was <13 mg.

#### Time spent in 24-hour movement behaviors

Wear time (days), time spent in moderate-to-vigorous physical activity (min/d), time spent in light physical activity (h/d), and total sedentary time (h/d) were derived using the Biobank Accelerometer Analysis Tool (https://github.com/OxWearables/biobankAccelerometerAnalysis, v7.1.1). This algorithm was evaluated in 152 adults under free-living conditions using a first-person still-image camera as the reference.^20^ Participants were excluded if the data could not be parsed, the device could not be calibrated, more than 1% of readings were ‘clipped’ (ie, fell outside ±8 g) before or after calibration, or if average acceleration was implausibly high (>100 mg). Participants were also excluded if they did not have sufficient wear time (≥3 days of data with coverage across every one-hour period of the 24-hour cycle). Non-wear time was defined as unbroken episodes of ≥60 minutes in which the *SD* of acceleration on each axis was <13 mg. To account for potential diurnal bias in wear time, recording interruptions and non-wear periods were imputed using the average values from the corresponding minute across the remaining valid days.

### Statistical analysis

We first reported the social, economic, and environmental characteristics of the analytic sample, presenting frequency and percentage for categorical variables, mean and *SD* for normally distributed continuous variables, and median and interquartile range (IQR) for non-normally distributed continuous variables. We also compared the social, economic, and environmental characteristics of individuals who agreed versus declined to wear an accelerometer, with associations assessed using the chi-squared test.

Next, for each accelerometer-based outcome (total wear time, overall daily step count, overnight sleep duration, time spent in moderate-to-vigorous physical activity, time spent in light physical activity, and total sedentary time), we reported descriptive statistics and examined variation by temporal characteristics (time of day and weekend day vs weekday). Differences by time of day (categorized as 00:00-05:59, 06:00-11:59, 12:00-17:59, or 18:00-23:59) were assessed using repeated measures analysis of variance (ANOVA) for normally distributed outcomes and the Friedman test for non-normally distributed outcomes. Differences between weekend days and weekdays were assessed using the paired *t*-test for normally distributed outcomes or the Wilcoxon signed-rank test for non-normally distributed outcomes.

Cross-sectional associations between accelerometer-based outcomes and social, economic, and environmental characteristics were examined using linear regression models. Table S2 shows the covariates included in the models for each characteristic of interest. Marginal means and 95% confidence intervals (CIs) were reported. Linear trends in accelerometer-based outcomes were assessed only for age groups and wealth quintiles, using separate models with age and wealth included as ordinal variables. Finally, to test the representativeness of our results, we conducted a sensitivity analysis restricted to core Wave 10 participants with valid accelerometry data, applying the core sample’s Wave 10 cross-sectional weights.

Statistical analyses were performed using RStudio (R version 4.4.1) (R Foundation for Statistical Computing, Vienna, Austria). Results have been reported according to the Strengthening the Reporting of Observational Studies in Epidemiology (STROBE) guidelines (Table S3).^21^

## Results

Between June 2021 and March 2023, 7482 adults aged ≥50 years consented to participate in Wave 10 of ELSA, of whom 5382 (71.9%) were invited to take part in the accelerometry substudy. Of those invited, 4354 (80.9%) agreed. The most commonly reported reasons for refusal were concerns about the monitor being uncomfortable (*n* = 296; 26.5%), the study being too time-consuming (*n* = 200; 17.9%), and being unable to wear the monitor at work (*n* = 118; 10.6%).

Figure S1 shows social, economic, and environmental disparities in the percentage of individuals who agreed to wear an accelerometer. Age, marital status, education, and wealth were all associated with agreement. Compared with individuals aged 50-59 years, those aged 60-79 years were more likely to agree, whereas those aged ≥ 80 years were less likely (Table S4). Individuals who were married, had intermediate or high education, or were in the top two wealth quintiles were also more likely to agree than their respective counterparts (Table S4).

Seventeen participants who agreed to wear an accelerometer were unable to do so due to injury, and a further six devices could not be activated. In total, 4331 participants were sent an accelerometer by post, of whom 4145 (95.7%) returned it. Ultimately, 3648 participants provided readable raw acceleration data and were included in the accelerometry subsample.

The social, economic, and environmental characteristics of the accelerometry subsample are shown in Table 1. Overall, 2033 (55.7%) were female, and the mean age was 68.5 ± 9.3 years. Most participants lived in an urban area (*n* = 2655; 73.0%), were married (*n* = 2478; 68.0%), had a high level of education (*n* = 1608; 44.7%), and were in the top two wealth quintiles (*n* = 1498; 49.4%). Most participants wore the accelerometer during autumn (*n* = 1155; 31.7%) or summer (*n* = 1054; 28.9%), while 858 (23.5%) completed accelerometer monitoring during spring and 581 (15.9%) during winter. The subsample was broadly similar to the overall Wave 10 sample.

**Table 1:** Social, economic, and environmental characteristics of the English Longitudinal Study of Aging (ELSA) accelerometry subsample versus the overall Wave 10 sample [*N* (%)].

Characteristics	Accelerometry subsample	Wave 10 sample
*N* (%)	3648	7482
**Sex**		
Male	1615 (44.3)	3360 (44.9)
Female	2033 (55.7)	4122 (55.1)
**Age (years)**		
Mean (*SD*)	68.5 (9.3)	68.3 (9.8)
50-59	748 (20.5)	1692 (22.6)
60-69	1224 (33.6)	2502 (33.4)
70-79	1206 (33.1)	2236 (29.9)
80−	470 (12.9)	1052 (14.1)
**Urbanicity^[^^*N*^ ^= 3637/7460]^**		
Urban	2655 (73.0)	5478 (73.4)
Rural	982 (27.0)	1982 (26.6)
**Marital status^[^^*N*^ ^= 3646/7477]^**		
Married	2478 (68.0)	4965 (66.4)
Not married	1168 (32.0)	2512 (33.6)
**Education^[^^*N*^ ^= 3600/7322]^**		
High	1608 (44.7)	3182 (43.5)
Intermediate	1251 (34.8)	2487 (34.0)
Low	741 (20.6)	1653 (22.6)
**Wealth^[^^*N*^ ^= 3032/6193]^**		
Quintile 5 (highest)	747 (24.6)	1406 (22.7)
Quintile 4 (high)	751 (24.8)	1470 (23.7)
Quintile 3 (intermediate)	638 (21.0)	1304 (21.1)
Quintile 2 (low)	500 (16.5)	1076 (17.4)
Quintile 1 (lowest)	396 (13.1)	937 (15.1)
**Season**		
Summer	1054 (28.9)	
Autumn	1155 (31.7)	
Winter	581 (15.9)	
Spring	858 (23.5)	

Sample sizes vary across characteristics owing to missing data for urbanicity, marital status, education, and wealth. The number of participants included in the accelerometry subsample and the Wave 10 sample is indicated by the table superscripts. Abbreviation: *SD*, standard deviation.

### Wear time

Among the 3648 participants in the accelerometry subsample, the median wear time was 6.6 days (IQR: 6.0-6.9; Table S5). Most participants (*n* = 2734; 75.0%) had >6 to ≤7 valid days of wear time. The remaining participants were distributed across lower wear time categories: 208 (5.7%) had >5 to ≤6 days, 110 (3.0%) had >4 to ≤5 days, 115 (3.2%) had >3 to ≤4 days, 103 (2.8%) had >2 to ≤3 days, 134 (3.7%) had >1 to ≤2 days, and 244 (6.7%) had ≤1 day.

Wear time was generally similar across social, economic, and environmental characteristics (Figure S2 and Table 2), although it was slightly higher among older and wealthier participants. On average, wear time was 3.2 h/d higher per 10-year increase in age (*p* for linear trend <.0001). Compared with the highest wealth quintile, wear time was 4.8 h/d higher in the high wealth quintile (*p *= .032) but 7.2 h/d lower in the lowest wealth quintile (*p *= .032); no other statistically significant differences were observed between wealth quintiles.

**Table 2:** Marginal mean (95% CI) wear time and 24-hour movement behaviors across social, economic, and environmental characteristics.

Characteristics	Wear time (days)	Step count (steps/d)	Sleep duration (h/night)	MVPA (min/d)	LPA (h/d)	SB (h/d)
*N*	3648	3161	3161	3161	3161	3161
**Sex**						
Male	5.8 (5.7-5.9)	8125 (7888-8362)	5.9 (5.8-6.0)	35.8 (34.0-37.6)	4.2 (4.1-4.3)	11.8 (11.7-12.0)
Female	5.7 (5.6-5.8)	7660 (7446-7875)	6.1 (6.0-6.1)	24.3 (22.7-25.9)	4.8 (4.7-4.9)	11.1 (11.0-11.2)
**Age (years)**						
50-59	5.5 (5.3-5.6)	9770 (9421-10119)	6.3 (6.2-6.4)	38.9 (36.3-41.5)	5.1 (4.9-5.2)	10.7 (10.5-10.8)
60-69	5.7 (5.6-5.8)	9160 (8897-9423)	6.2 (6.1-6.3)	40.2 (38.2-42.2)	4.7 (4.5-4.8)	11.1 (11.0-11.3)
70-79	5.8 (5.7-5.9)	7382 (7123-7642)	6.0 (5.9-6.1)	26.3 (24.4-28.3)	4.4 (4.3-4.5)	11.6 (11.5-11.7)
80-	5.9 (5.7-6.1)	5259 (4848-5671)	5.4 (5.3-5.6)	14.7 (11.6-17.8)	3.8 (3.6-4.0)	12.5 (12.2-12.7)
**Education^[^^*N*^ ^= 3600/3123]^**						
High	5.7 (5.6-5.8)	8348 (8109-8586)	6.1 (6.0-6.1)	33.7 (31.9-35.5)	4.5 (4.4-4.6)	11.4 (11.3-11.5)
Intermediate	5.8 (5.7-5.9)	7904 (7636-8172)	6.0 (5.9-6.1)	28.9 (26.9-30.9)	4.6 (4.5-4.7)	11.4 (11.3-11.6)
Low	5.7 (5.5-5.8)	6939 (6601-7277)	5.8 (5.7-5.9)	24.0 (21.4-26.5)	4.3 (4.2-4.5)	11.6 (11.5-11.8)
**Marital status^[^^*N*^ ^= 3598/3122]^**						
Married	5.8 (5.7-5.8)	7998 (7790-8205)	6.0 (6.0-6.1)	29.6 (28.0-31.2)	4.6 (4.5-4.6)	11.3 (11.2-11.4)
Not married	5.7 (5.6-5.8)	7238 (6963-7512)	5.8 (5.7-5.9)	27.6 (25.5-29.7)	4.3 (4.2-4.4)	11.8 (11.6-11.9)
**Wealth^[^^*N*^ ^= 2999/2616]^**						
Highest	5.7 (5.5-5.8)	8564 (8202-8926)	6.0 (5.9-6.2)	34.9 (32.2-37.6)	4.6 (4.4-4.7)	11.2 (11.1-11.4)
High	5.9 (5.7-6.0)	8038 (7698-8377)	6.1 (6.0-6.2)	32.4 (29.8-34.9)	4.5 (4.4-4.6)	11.3 (11.1-11.5)
Intermediate	5.8 (5.6-5.9)	7118 (6765-7471)	5.9 (5.8-6.0)	25.7 (23.1-28.4)	4.4 (4.3-4.6)	11.6 (11.4-11.8)
Low	5.8 (5.7-6.0)	7146 (6754-7539)	5.8 (5.7-5.9)	25.9 (23.0-28.9)	4.3 (4.1-4.5)	11.8 (11.6-12.0)
Lowest	5.4 (5.2-5.6)	6831 (6364-7298)	5.6 (5.4-5.8)	20.3 (16.8-23.8)	4.4 (4.2-4.6)	12.0 (11.8-12.2)
**Urbanicity^[^^*N*^ ^= 2989/2606]^**						
Urban	5.7 (5.6-5.8)	7360 (7153-7567)	5.9 (5.8-5.9)	26.8 (25.3-28.4)	4.4 (4.3-4.5)	11.6 (11.5-11.7)
Rural	5.8 (5.6-5.9)	8149 (7818-8480)	5.9 (5.8-6.1)	31.4 (28.9-33.8)	4.5 (4.4-4.6)	11.4 (11.3-11.6)

Sample sizes vary across models owing to missing data for education, marital status, wealth, and urbanicity. The number of participants included in each wear time and 24-hour movement behavior analysis is indicated by the table superscripts.

The model for sex was adjusted for season of accelerometer wear and age group.

The model for age group was adjusted for season of accelerometer wear and sex.

The model for education was adjusted for season of accelerometer wear, age group, sex, and education.

The model for wealth was adjusted for season of accelerometer wear, age group, sex, education, and marital status.

The model for urbanicity was adjusted for season of accelerometer wear, age group, sex, education, marital status, and wealth.

Abbreviations: 95% CI, 95% confidence interval; MVPA, moderate-to-vigorous physical activity; LPA, light physical activity; SB, sedentary behavior.

### 24-hour movement behaviors

Among the accelerometry subsample, 3164 participants (86.7%) had sufficient wear time to assess 24-hour movement behaviors. A further three participants were excluded because the device could not be calibrated, more than 1% of readings were ‘clipped’ (ie, fell outside the device’s dynamic range of ±8 g) before or after calibration, or one of the accelerometer-based outcomes was missing. This resulted in a final analytic sample of 3161 participants (Figure 1).

**Figure 1:**
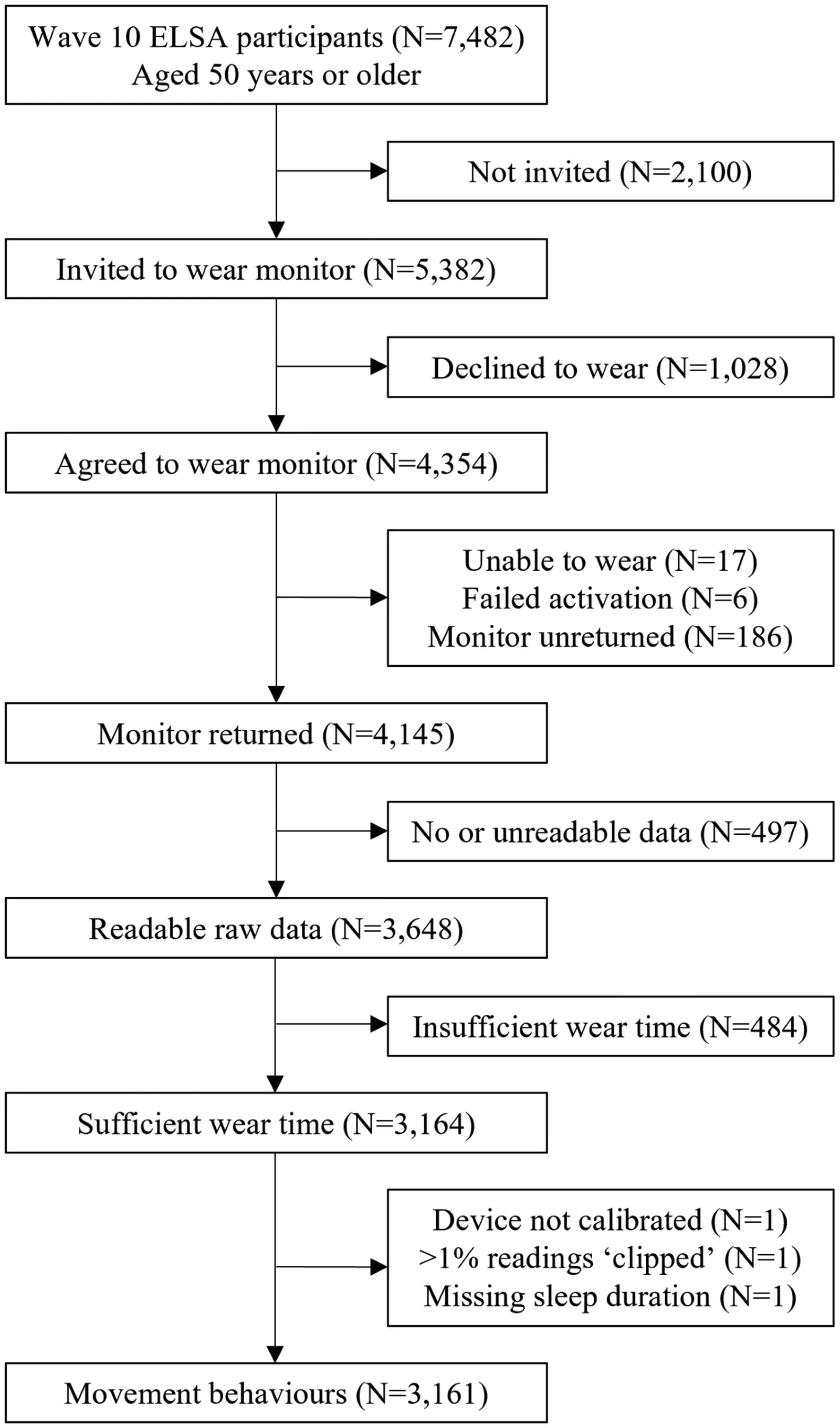
Participant flow chart of the English Longitudinal Study of Aging (ELSA) accelerometer data collection from 2021-2023.

#### Overall daily step count

Among the 3161 participants with valid 24-hour movement behavior data, the median overall daily step count was 7658 steps/day (IQR: 4892 to 10 779 steps/d; Table S5). Figure 2A and Table 2 show social, economic, and environmental disparities in overall daily step count. Step count was 465 steps/day lower in females than in males (*p *= .0027), 789 steps/d higher in rural compared with urban participants (*p *< .0001), and 760 steps/d lower in unmarried compared with married participants (*p *< .0001). Step count was, on average, 1504 steps/d lower per 10-year increase in age (*p* for linear trend <.0001). Compared with participants with a high level of education, step count was 444 steps/d lower in those with an intermediate level (*p *= .011) and 1409 steps/d lower in those with a low level (*p *< .0001). Finally, step count was, on average, 433 steps/d lower per one quintile lower wealth (*p* for linear trend <.0001).

**Figure 2:**
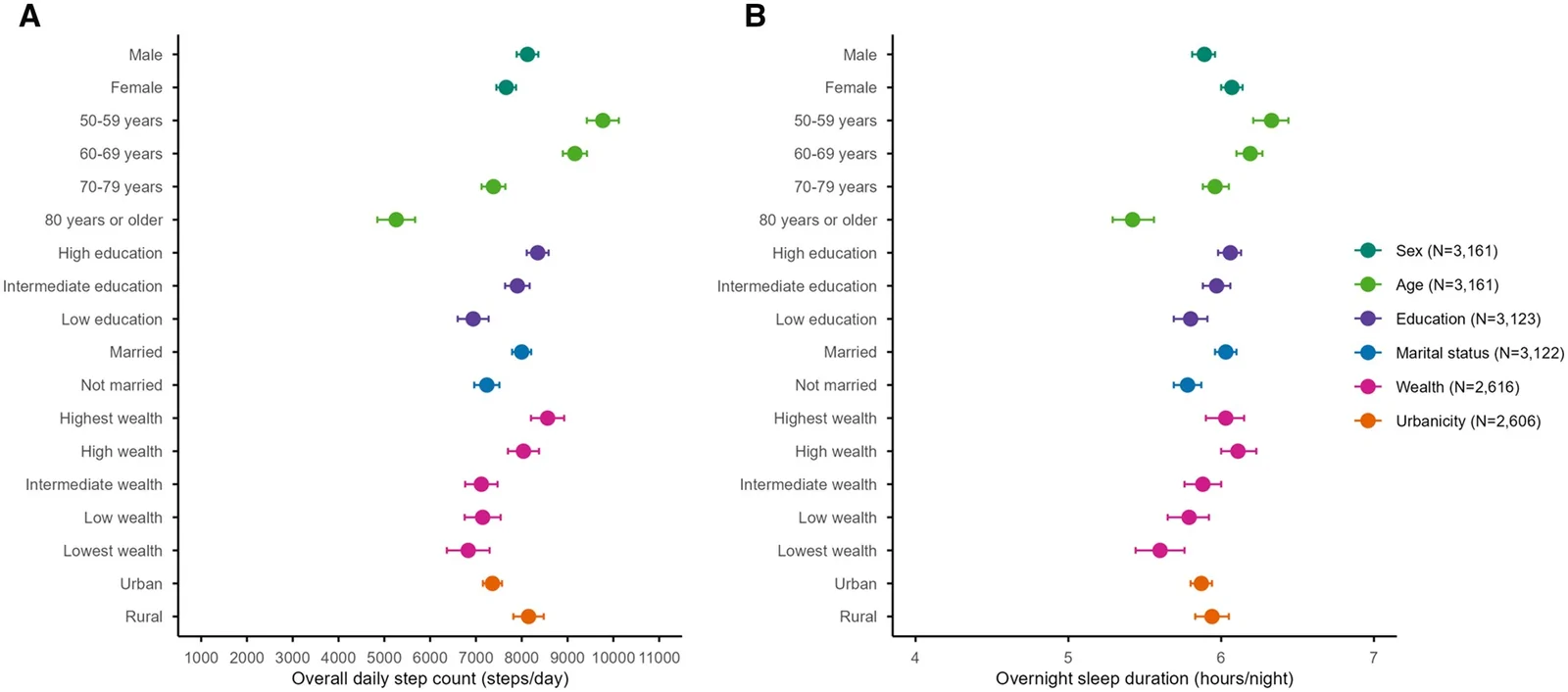
Marginal mean (95% CI) overall daily step count (A) and overnight sleep duration (B) across social, economic, and environmental characteristics (*N* = 3161). The model for sex was adjusted for season of accelerometer wear and age group. The model for age group was adjusted for season of accelerometer wear and sex. The model for education was adjusted for season of accelerometer wear, age group, sex, and education. The model for wealth was adjusted for season of accelerometer wear, age group, sex, education, and marital status. The model for urbanicity was adjusted for season of accelerometer wear, age group, sex, education, marital status, and wealth. 95% CI, 95% confidence interval.

#### Overnight sleep duration

The median overnight sleep duration was 6.3 h/night (IQR: 5.3-7.0 hours/night; Table S5). Figure 2B and Table 2 show social, economic, and environmental disparities in overnight sleep duration. Sleep duration was 12 minutes/night longer in females than in males (*p *= .00039) and 12 minutes/night shorter in unmarried participants compared with married participants (*p *< .0001). Sleep duration was, on average, 18 minutes/night shorter per 10-year increase in age (*p* for linear trend < .0001). Compared with participants with a high level of education, sleep duration was 18 minutes/night shorter in those with a low level (*p *= .00021) but not significantly different in those with an intermediate level (*p *= .15). Sleep duration was, on average, 6 minutes/night shorter per one-quintile lower wealth (*p* for linear trend < .0001). There was no statistically significant difference in sleep duration between rural and urban participants (*p *= .26).

#### Time spent in moderate-to-vigorous physical activity

The median amount of time spent in moderate-to-vigorous physical activity was 20.6 min/d (IQR: 5.8-45.0 min/d; Table S5). Figure S3 and Table 2 show social, economic, and environmental disparities in time spent in moderate-to-vigorous physical activity. Moderate-to-vigorous physical activity was 12 min/d lower in females than in males (*p *< .0001) and 5 min/d higher in rural compared with urban participants (*p *= .0010). Moderate-to-vigorous physical activity was, on average, 8 min/d lower per 10-year older age (*p* for linear trend < .0001). Compared with participants with a high level of education, moderate-to-vigorous physical activity was 5 min/d lower in those with an intermediate level (*p *= .00023) and 10 min/d lower in those with a low level (*p *< .0001). Moderate-to-vigorous physical activity was, on average, 4 min/d lower per one-quintile lower wealth (*p* for linear trend < .0001). There was no statistically significant difference in moderate-to-vigorous physical activity between married and unmarried participants (*p *= .12).

#### Time spent in light physical activity

The median amount of time spent in light physical activity was 4.5 h/d (IQR: 3.3-5.8 h/d; Table S5). Figure S4 and Table 2 show social, economic, and environmental disparities in time spent in light physical activity. Light physical activity was 36 min/d higher in females than in males (*p *< .0001) and 18 min/d lower in unmarried participants compared with married participants (*p *= .00047). Light physical activity was, on average, 26 min/d lower per 10-year older age (*p* for linear trend <.0001) and 3 min/d lower per one-quintile lower wealth (*p* for linear trend = .0093). Compared with participants with a high level of education, light physical activity was 12 min/d lower in those with a low level (*p *= .045) but not significantly different in those with an intermediate level (*p *= .12). There was no statistically significant difference in light physical activity between rural and urban participants (*p *= .21).

#### Total sedentary time

The mean total sedentary time was 11.3 h/d (*SD*: 2.3 h/d; Table S5). Figure S5 and Table 2 show social, economic, and environmental disparities in total sedentary time. Total sedentary time was 42 min/d lower in females than in males (*p *< .0001) and 30 min/d higher in unmarried participants compared with married participants (*p *< .0001). Total sedentary time was, on average, 36 min/d higher per 10-year older age (*p* for linear trend <.0001). Compared with participants with a high level of education, total sedentary time was 12 min/d higher in those with a low level (*p *= .028) but not significantly different in those with an intermediate level (*p *= .98). Total sedentary time was, on average, 12 min/d higher per one-quintile lower wealth (*p* for linear trend <.0001). There was no statistically significant difference in total sedentary time between rural and urban participants (*p *= .065).

#### Temporal patterns

Table S6 shows temporal disparities in wear time and 24-hour movement behaviors. Step count, light physical activity, and total sedentary time were lower on weekend days than on weekdays, whereas sleep duration was longer on weekends. There were no statistically significant differences in wear time or moderate-to-vigorous physical activity between weekdays and weekend days.

All accelerometer-based outcomes differed by time of day (Figures S6-S11). Wear time, step count, moderate-to-vigorous physical activity, and light physical activity were highest between 12:00-17:59 and lowest between 00:00-05:59. Total sedentary time was also lowest between 00:00-05:59 but highest between 18:00-23:59. In contrast, sleep duration was longest between 00:00-05:59 and shortest between 12:00-17:59.

### Sensitivity analysis

Social, economic, and environmental disparities were largely unchanged when the core Wave 10 cross-sectional weights were applied to the restricted sample (Table S7).

## Discussion

In this accelerometry substudy of the nationally representative ELSA prospective cohort, response (81%) and adherence (87%) rates were high among English adults aged ≥50 years. Older and wealthier participants wore the accelerometer for longer. Compared with males, females accumulated fewer steps, engaged in less moderate-to-vigorous physical activity, and had more sedentary time, but more light activity and longer sleep. Participants who were older, unmarried, or had lower education or wealth were less active, more sedentary, and slept less than their respective counterparts. Rural participants were more active than urban participants. Finally, participants slept longer on weekend days than on weekdays, resulting in less time spent in most waking behaviors.

In terms of socioeconomic disparities in waking behaviors, both lower education and lower wealth were associated with lower physical activity and higher sedentary time. This contrasts with findings from other accelerometry cohorts, which have either reported no significant socioeconomic differences in waking behaviours^10^^,^^13^^,^^18^^,^^20^ or observed higher overall physical activity among participants with lower education or wealth, primarily reflecting more physically demanding (ie, blue-collar) occupations.^11^^,^^15^ Age differences between study populations may partly explain these discrepancies. Participants in our study were older and therefore more likely to be retired, reducing the contribution of occupational physical activity and increasing the relative importance of leisure-time physical activity. Leisure-time physical activity tends to be lower among participants with lower education or wealth due to barriers such as limited energy or financial resources.^11^^,^^15^ Socioeconomic disparities in waking behaviors may contribute to health inequalities, reinforcing disadvantaged adults as a key target group for interventions to increase physical activity and reduce sedentary time.

Regarding sex differences in physical activity and sedentary time, our findings are consistent with findings from UKB,^7^^,^^18^^,^^20^ NHANES,^15^ FinHealth,^10^ ORISCAV-LUX 2,^11^ and CKB,^13^ where overall physical activity was higher in females than in males. Previous research suggests that this difference may be partly explained by greater engagement in light-intensity activities such as housework and childcare.^22^ In contrast, findings from the Brazilian Study on Nutrition and Health showed a different pattern: females accumulated fewer steps and less moderate-to-vigorous physical activity than males, but this was not offset by a higher ratio of light activity to sedentary behavior.^23^ This discrepancy may reflect the use of a waist-worn accelerometer, which is less effective at capturing activities of daily living, or the younger age of the participants. Alternatively, it may reflect differences in socioeconomic patterns of 24-hour movement behaviors between low- and middle-income and high-income countries. Given the limited number of accelerometry studies in low- and middle-income countries, further research is warranted.

In terms of sleep, we found that participants who were male, older, unmarried, or had lower education or wealth had shorter overnight sleep duration. To our knowledge, only UKB and CKB have examined socioeconomic disparities in device-measured sleep.^13^^,^^19^^,^^20^ Consistent with our findings, UKB reported shorter overnight sleep duration in males but found a positive rather than inverse association with age.^19^ This discrepancy likely reflects the younger age range in UKB (40-79 years) compared with ELSA (≥50 years), meaning most UKB participants had not yet reached the age at which sleep decline is most pronounced. UKB reported no other socioeconomic differences in overnight sleep duration.^19^ Although our findings highlight potential key target groups for sleep interventions, further research is needed in other large-scale international accelerometry cohorts.

### Strengths and limitations

A key strength of this study is that the analytic sample was drawn from the nationally representative ELSA cohort, enhancing the generalizability of our findings compared with other wrist-worn accelerometry cohorts such as UKB,^7^ the British Whitehall II study,^8^ the Rotterdam study,^12^ CKB,^13^ and the Pelotas birth cohorts.^14^ Furthermore, unlike nationally representative cohorts from the US,^9^ Finland,^10^ and Luxembourg,^11^ our study focused specifically on older adults—a key target group for 24-hour movement behavior interventions given their lower levels of physical activity, higher levels of sedentary time, and shorter sleep duration. However, because not all ELSA participants were invited to participate in the accelerometry substudy, and because selective participation and attrition over successive ELSA waves may have affected the composition of the sample, the analytic sample may not be fully representative of the broader English population. Although applying the Wave 10 core sample cross-sectional weights to the analytic sample produced materially similar results, suggesting that potential selection and participation biases did not substantially influence our findings.

Another key strength is that wrist-worn raw acceleration data were processed using three well-validated, open-source machine learning algorithms to derive meaningful movement behaviors across the full 24-hour spectrum. While previous studies have examined socioeconomic disparities in device-measured physical activity, relatively few have investigated such differences in device-measured sleep. The free-living validation study used to derive estimates of time spent in 24-hour movement behaviors included a single gold-standard measure for both physical activity and sedentary behavior (ie, a first-person still-image camera), but it did not include a gold-standard measure of sleep (ie, PSG). Therefore, it is possible that naps were misclassified as sedentary time, which we acknowledge as a limitation. However, in a Chinese population—where midday napping is common—there is some evidence suggesting that the algorithm may capture napping behavior, as accelerometer-derived sleep time increased between 12:00 and 15:00.^13^ Furthermore, our primary sleep measure—overnight sleep duration—was derived using a separate machine learning algorithm, which was validated against a gold-standard measure of sleep (ie, laboratory-based PSG). Sleep staging data were not analyzed in the current study because, in the absence of additional physiological signals such as electrocardiography (ECG) and photoplethysmography (PPG), our algorithm—which uses features extracted solely from raw triaxial acceleration signals—tends to underestimate REM sleep and overestimate NREM sleep.^19^ All three algorithms were developed using data from younger adults.^18–20^ While the observed social, economic, and environmental disparities, together with the expected temporal patterns, provide some support for the face validity of these algorithms, further validation in older adults against ground-truth measures is needed.

A final key strength of our study is the examination of disparities across a broader set of factors through which 24-hour movement behaviors both reflect the social determinants of health and contribute to social inequalities in health. While previous studies primarily focused on age, sex, and education, we also examined marital status, wealth, and urbanicity. This broader perspective enabled the identification of additional population groups who may benefit from targeted interventions, specifically unmarried, economically disadvantaged, and urban participants.

Due to the COVID-19 pandemic, data collection was conducted via telephone or video call rather than in person, with accelerometers distributed by post. Nevertheless, both response and adherence rates were high. A limitation of this study is that participants wore the accelerometer for a single seven-day period, which may not fully capture habitual 24-hour movement behaviors across the year. However, a recent UKB analysis reported moderate-to-good agreement between measurements taken 3-4 years apart, ranging from 58% to 75% across different 24-hour movement behaviors.^24^ Another limitation is that wrist-worn accelerometers are limited in their ability to detect certain postures or efforts requiring higher energy expenditure, such as carrying loads. Nevertheless, the increasing preference for wrist placement in recent studies, largely driven by higher wear time,^25^ enables ELSA accelerometer data to be pooled and/or compared with other large-scale epidemiological cohorts from the UK,^7^^,^^8^ the US,^9^ Europe,^10–12^ China,^13^ and Brazil.^14^

## Conclusion

The current accelerometry substudy is, to our knowledge, the first study from a population-based prospective cohort of older adults to apply open-source, well-validated machine learning algorithms to raw triaxial acceleration data to measure movement behaviors across the full 24-hour spectrum, including physical activity, sedentary behavior, and sleep. It highlights older, unmarried, or socioeconomically disadvantaged adults as key target groups for interventions to increase physical activity, reduce sedentary time, and optimize sleep in later life. Adults living in urban areas may also represent an important target population for physical activity interventions. With follow-up data, the nationally representative ELSA cohort will be well positioned to clarify how 24-hour movement behaviors influence healthy aging, reflect underlying social determinants of health, and contribute to social inequalities in health. These data can also be pooled with, or compared across, other large-scale epidemiological cohorts that have collected or will collect wrist-worn raw triaxial acceleration data, thereby strengthening the evidence base to inform future international physical activity guidelines.

## Supplementary material

Supplementary material is available at *The Journals of Gerontology, Series A: Biological Sciences and Medical Sciences* online.

## Funding

L.B. was supported by the Economic and Social Research Council (ES/T014091/1) and the Wellcome Trust (223100/Z/21/Z). M.B. was supported by the Economic and Social Research Council (ES/T014091/1). A.D.’s research team was supported by grants from the Wellcome Trust (223100/Z/21/Z and 227093/Z/23/Z), Novo Nordisk, Swiss Re, Boehringer Ingelheim, the NIH Oxford-Cambridge Scholars Program, EPSRC Center for Doctoral Training in Health Data Science (EP/S02428X/1), the British Heart Foundation Center of Research Excellence (RE/18/3/34214), and the Danish National Research Foundation, which supports the Pioneer Center for SMARTbiomed. The funders had no role in study design, data collection, analysis, interpretation, writing, or the decision to submit the manuscript for publication. A.D. has received research funding from the Wellcome Trust, Novo Nordisk, Swiss Re, Boehringer Ingelheim, the British Heart Foundation, Health Data Research UK, and Google (supporting research into wearables phenotyping) and has received donations of equipment from Swiss Re and Google.

## Conflicts of interest

A.D. also receives software-license royalties from GlaxoSmithKline and serves on advisory boards for the EU iPROLEPSIS and EU IMI IDEA-FAST projects. All other authors declare no conflicts.

## Supplementary Material

glag197_Supplementary_Data

## Data Availability

Data from ELSA are available to researchers upon application. The interview data used in this study can be accessed free of charge via the UK Data Service (https://ukdataservice.ac.uk/) following registration and agreement to the data’s end user license. The accelerometer data used in this study will be made available through the UK Data Service in due course.
