# Supplementary material for: Social, economic, and environmental disparities in device-measured 24-hour movement behaviors in a nationally representative cohort of older English adults
Source: J Gerontol A Biol Sci Med Sci. 2026 Aug 19;81(11):glag197. doi: 10.1093/gerona/glag197 (PMC13690001; doi:10.1093/gerona/glag197)
Supplement: Supplementary file 1 [file glag197_supplementary_data.docx]

**Social, economic, and environmental disparities in device-measured 24-hour movement behaviours in a nationally representative cohort of older English adults**

**Supplementary material**

**Table of contents**

Pages 2-3	Table A1. Derivation of social, economic, and environmental factors.

Page 4	Table A2. Covariates included in the models for each characteristic of interest.

Page 5	Table A3. STROBE statement checklist.

Page 6	Table A4. Percentage of individuals who agreed to wear an accelerometer across social, economic, and environmental characteristics (N = 5,382).

Page 7	Table A5. Mean or median (SD or IQR) wear time and 24-hour movement behaviours across social, economic, and environmental characteristics.

Page 8	Table A6. Mean or median (SD or IQR) wear time and 24-hour movement behaviours across temporal characteristics.

Page 9	Table A7. Marginal mean (95% CI) wear time and 24-hour movement behaviours across social, economic, and environmental characteristics, after applying the core Wave 10 cross-sectional weights to the restricted sample^a^.

Page 10	Figure A1. Percentage of individuals who agreed to wear an accelerometer across social, economic, and environmental characteristics (N = 5,382).

Page 11	Figure A2. Marginal mean (95% CI) wear time across social, economic, and environmental characteristics (N = 3,648).

Page 12	Figure A3. Marginal mean (95% CI) time spent in moderate-to-vigorous physical activity across social, economic, and environmental characteristics (N = 3,161).

Page 13	Figure A4. Marginal mean (95% CI) time spent in light physical activity across social, economic, and environmental characteristics (N = 3,161).

Page 14	Figure A5. Marginal mean (95% CI) total sedentary time across social, economic, and environmental characteristics (N = 3,161).

Page 15	Figure A6. 24-hour profile of wear time by age group (N = 3,648).

Page 16	Figure A7. 24-hour profile of step count by age group (N = 3,161).

Page 17	Figure A8. 24-hour profile of sleep duration by age group (N = 3,161).

Page 18	Figure A9. 24-hour profile of moderate-to-vigorous physical activity by age group (N = 3,161).

Page 19	Figure A10. 24-hour profile of light physical activity by age group (N = 3,161).

Page 20	Figure A11. 24-hour profile of total sedentary time by age group (N = 3,161).

**Table A1. Derivation of social, economic, and environmental factors.**

| **Factor** | **Original categories** | **Derived categories** | **Notes** |
| --- | --- | --- | --- |
| Season | September | Autumn | Season was defined based on the month at the end of the accelerometer wear period. |
|  | October |  |  |
|  | November |  |  |
|  | December | Winter |  |
|  | January |  |  |
|  | February |  |  |
|  | March | Spring |  |
|  | April |  |  |
|  | May |  |  |
|  | June | Summer |  |
|  | July |  |  |
|  | August |  |  |
| Sex | Male | Male |  |
|  | Female | Female |  |
| Age | Continuous age in years | 50-59 |  |
|  |  | 60-69 |  |
|  |  | 70-79 |  |
|  |  | ≥80 |  |
| Marital status | Married, first and only marriage or a civil partner in a registered same-sex Civil Partnership | Married |  |
|  | Remarried, second or later marriage |  |  |
|  | Single, that is never married and never registered in a same-sex Civil Partnership | Not married |  |
|  | Separated, but still legally married or (spontaneous only) in a same-sex Civil Partnership |  |  |
|  | Divorced or (spontaneous only) formerly in a same-sex Civil Partnership |  |  |
|  | Widowed or (spontaneous only) a surviving civil partner from a same-sex Civil Partnership |  |  |
| Education | Degree/degree level qualification (including higher degree) | High education |  |
|  | Teaching qualification |  |  |
|  | Nursing qualifications SRN, SCM, SEN, RGN, RM, RHV, Midwife |  |  |
|  | HNC/HND, BEC/TEC Higher, BTEC Higher/SCOTECH Higher | Intermediate education |  |
|  | ONC/OND/BEC/TEC/BTEC not higher |  |  |
|  | City and Guilds Full Technological Certificate |  |  |
|  | City and Guilds Advanced/Final Level |  |  |
|  | City and Guilds Craft/Ordinary Level |  |  |
|  | A-levels/Higher School Certificate |  |  |
|  | AS Level |  |  |
|  | SLC/SCE/SUPE at Higher Grade or Certificate of Sixth Year Studies |  |  |
|  | Recognised Trade Apprenticeship completed |  |  |
|  | Clerical or Commercial Qualification (e.g. typing/book-keeping/commerce) |  |  |

|  | O-level passes taken in 1975 or earlier | Low education |  |
| --- | --- | --- | --- |
|  | O-level passes taken after 1975 GRADES A-C |  |  |
|  | O-level passes taken after 1975 GRADES D-E |  |  |
|  | GCSE GRADES A-C |  |  |
|  | GCSE GRADES D-G |  |  |
|  | CSE GRADE 1/SCE BANDS A-C/Standard Grade LEVEL 1-3 |  |  |
|  | CSE GRADES 2-5/SCE Ordinary BANDS D-E |  |  |
|  | CSE Ungraded |  |  |
|  | SLC Lower |  |  |
|  | SUPE Lower or Ordinary |  |  |
|  | School Certificate or Matric |  |  |
|  | NVQ Level 5 |  |  |
|  | NVQ Level 4 |  |  |
|  | NVQ Level 3/Advanced level GNVQ |  |  |
|  | NVQ Level 2/Intermediate level GNVQ |  |  |
|  | NVQ Level 1/Foundation level GNVQ |  |  |
| Wealth | Net total non-housing wealth (savings, investments, physical wealth, and housing wealth minus financial and mortgage debt) | Quintiles (1-5) | Wealth quintiles were derived from the Wave 10 distribution of net total non-housing wealth. The observed wealth ranges within each quintile were: Q1 ≤£1,600; Q2 £1,665-£21,000; Q3 £21,020-£81,200; Q4 £81,499-£280,000; and Q5 ≥£280,020. For participants with missing Wave 10 data, the most recent available wealth quintile from Waves 9-1 was used. |
| Urbanicity | Major conurbation | Urban | The ONS Rural-Urban Classification (2011) was linked to participants’ residential postcode via Census Output Areas and used to derive urban/rural status. |
|  | Minor conurbation |  |  |
|  | City and town |  |  |
|  | City or town in sparse settings |  |  |
|  | Town or fringe | Rural |  |
|  | Town or fringe in sparse settings |  |  |
|  | Village |  |  |
|  | Village in sparse settings |  |  |
|  | Hamlets and isolated dwellings |  |  |
|  | Hamlets and isolated dwellings in a sparse setting |  |  |

**Table A2. Covariates included in the models for each characteristic of interest.**

| **Characteristic** | **Covariates** |
| --- | --- |
| Sex | Season of accelerometer wear and age |
| Age | Season of accelerometer wear and sex |
| Education | Season of accelerometer wear, age, and sex |
| Marital status | Season of accelerometer wear, age, sex, and **education** |
| Wealth | Season of accelerometer wear, age, sex, education, and **marital status** |
| Urbanicity | Season of accelerometer wear, age, sex, education, marital status, and **wealth** |

**Table A3. STROBE statement checklist.**

|  | **Item number** | **Recommendation** | **Page number** |
| --- | --- | --- | --- |
| **Title and abstract** | 1 | (*a*) Indicate the study’s design with a commonly used term in the title or the abstract | 1,2 |
|  |  | (*b*) Provide in the abstract an informative and balanced summary of what was done and what was found | 2 |
| **Introduction** | | | |
| Background/rationale | 2 | Explain the scientific background and rationale for the investigation being reported | 3-4 |
| Objectives | 3 | State specific objectives, including any prespecified hypotheses | 4 |
| **Methods** | | | |
| Study design | 4 | Present key elements of study design early in the paper | 4 |
| Setting | 5 | Describe the setting, locations, and relevant dates, including periods of recruitment, exposure, follow-up, and data collection | 4 |
| Participants | 6 | (*a*) Give the eligibility criteria, and the sources and methods of selection of participants. Describe methods of follow-up | 4 |
|  |  | (*b*) For matched studies, give matching criteria and number of exposed and unexposed | N/A |
| Variables | 7 | Clearly define all outcomes, exposures, predictors, potential confounders, and effect modifiers. Give diagnostic criteria, if applicable | 5,6 |
| Data sources/measurement | 8* | For each variable of interest, give sources of data and details of methods of assessment (measurement). Describe comparability of assessment methods if there is more than one group | 5-7 |
| Bias | 9 | Describe any efforts to address potential sources of bias | 4-8 |
| Study size | 10 | Explain how the study size was arrived at | 9,10  Fig 1 |
| Quantitative variables | 11 | Explain how quantitative variables were handled in the analyses. If applicable, describe which groupings were chosen and why | 5-7  Table A1 |
| Statistical methods | 12 | (*a*) Describe all statistical methods, including those used to control for confounding | 8  Table A2 |
|  |  | (*b*) Describe any methods used to examine subgroups and interactions | N/A |
|  |  | (*c*) Explain how missing data were addressed | 6 |
|  |  | (*d*) If applicable, explain how loss to follow-up was addressed | N/A |
|  |  | (*e*) Describe any sensitivity analyses | 8 |
| **Results** | | |  |
| Participants | 13* | *(a)* Report numbers of individuals at each stage of study – e.g., numbers potentially eligible, examined for eligibility, confirmed eligible, included in the study, completing follow-up, and analysed | 9-10  Fig 1 |
|  |  | *(b)* Give reasons for non-participation at each stage | 9-10 |
|  |  | *(c)* Consider use of a flow diagram | Fig 1 |
| Descriptive data | 14* | *(a)* Give characteristics of study participants (e.g., demographic, clinical, social) and information on exposures and potential confounders | 9  Table 1 |
|  |  | *(b)* Indicate number of participants with missing data for each variable of interest | Table 1 |
|  |  | *(c)* Summarise follow-up time (e.g., average and total amount) | N/A |
| Outcome data | 15* | Report numbers of outcome events or summary measures over time | N/A |
| Main results | 16 | (*a*) Give unadjusted estimates and, if applicable, confounder-adjusted estimates and their precision (e.g., 95% confidence interval). Make clear which confounders were adjusted for and why they were included | 9-12  Table 2  Table A2  Table A5 |
|  |  | (*b*) Report category boundaries when continuous variables were categorised | Table 2 |
|  |  | (*c*) If relevant, consider translating estimates of relative risk into absolute risk for a meaningful time period | N/A |
| Other analyses | 17 | Report other analyses done—e.g., analyses of subgroups and interactions, and sensitivity analyses | 13  Table A7 |
| **Discussion** |  |  |  |
| Key results | 18 | Summarise key results with reference to study objectives | 13 |
| Limitations | 19 | Discuss limitations of the study, taking into account sources of potential bias or imprecision. Discuss both direction and magnitude of any potential bias | 14-16 |
| Interpretation | 20 | Give a cautious overall interpretation of results considering objectives, limitations, multiplicity of analyses, results from similar studies, and other relevant evidence | 13-14 |
| Generalisability | 21 | Discuss the generalisability (external validity) of the study results | 14-15 |
| **Other information** |  |  |  |
| Funding | 22 | Give the source of funding and the role of the funders for the present study and, if applicable, for the original study on which the present article is based | 17 |

* Information given separately for exposed and unexposed groups.

**Table A4. Percentage of individuals who agreed to wear an accelerometer across social, economic, and environmental characteristics (N = 5,382).**

| **Characteristics** | **Agreed to wear accelerometer [N (%)]** | ***P*-value** |
| --- | --- | --- |
| Overall | 4354 (80.9) |  |
| Sex |  | 0.39 |
| Male | 1907 (80.4) |  |
| Female | 2447 (81.3) |  |
| Age (years) |  | **<0.0001** |
| 50-59 | 944 (79.7) |  |
| 60-69 | 1472 (83.1) |  |
| 70-79 | 1387 (83.2) |  |
| 80- | 551 (72.7) |  |
| Urbanicity ^[N=5363]^ |  | 0.10 |
| Urban | 3177 (80.4) |  |
| Rural | 1163 (82.4) |  |
| Marital status ^[N=5377]^ |  | **0.0033** |
| Married | 2909 (82.1) |  |
| Not married | 1443 (78.7) |  |
| Education ^[N=5296]^ |  | **<0.0001** |
| High | 1886 (82.3) |  |
| Intermediate | 1509 (82.7) |  |
| Low | 893 (75.7) |  |
| Wealth ^[N=4528]^ |  | **0.00050** |
| Quintile 5 (highest) | 868 (83.7) |  |
| Quintile 4 (high) | 880 (82.2) |  |
| Quintile 3 (intermediate) | 753 (79.7) |  |
| Quintile 2 (low) | 619 (77.3) |  |
| Quintile 1 (lowest) | 519 (76.9) |  |

**Table A5. Mean or median (SD or IQR) wear time and 24-hour movement behaviours across social, economic, and environmental characteristics.**

| **Characteristics** | **Wear time**  **(days)** | **Step count**  **(steps/day)** | **Sleep duration**  **(hours/night)** | **MVPA**  **(minutes/day)** | **LPA**  **(hours/day)** | **SB**  **(hours/day)** |
| --- | --- | --- | --- | --- | --- | --- |
| N | 3648 | 3161 | 3161 | 3161 | 3161 | 3161 |
| Overall | 6.6  (6.0-6.9) | 7658  (4892-10779) | 6.3  (5.3-7.0) | 20.6  (5.8-45.0) | 4.5  (3.3-5.8) | 11.3 ± 2.3 |
| Sex |  |  |  |  |  |  |
| Male | 6.6  (6.1-7.0) | 7761  (4972-11018) | 6.2  (5.2-6.9) | 25.9  (8.6-55.1) | 4.1  (3.0-5.3) | 11.8 ± 2.3 |
| Female | 6.5  (5.8-6.9) | 7536  (4839-10506) | 6.4  (5.4-7.0) | 17.1  (4.7-38.7) | 4.8  (3.6-6.1) | 11.0 ± 2.2 |
| Age (years) |  |  |  |  |  |  |
| 50-59 | 6.5  (5.4-6.9) | 9217  (6683-12503) | 6.5  (5.8-7.1) | 29.9  (12.0-53.4) | 5.0  (3.8-6.5) | 10.6 ± 2.2 |
| 60-69 | 6.6  (6.0-6.9) | 8703  (5580-11967) | 6.4  (5.6-7.1) | 28.6  (10.0-57.9) | 4.6  (3.5-5.9) | 11.1 ± 2.2 |
| 70-79 | 6.6  (6.2-6.9) | 7076  (4664-9691) | 6.2  (5.3-7.0) | 17.3  (4.9-38.4) | 4.4  (3.2-5.6) | 11.6 ± 2.2 |
| 80- | 6.6  (6.2-6.9) | 4970  (2626- 7340) | 5.6  (4.4-6.6) | 7.1  (1.2-18.3) | 3.7  (2.7-5.0) | 12.4 ± 2.3 |
| Urbanicity ^[N=3637/3150]^ |  |  |  |  |  |  |
| Urban | 6.5  (5.7-6.9) | 7391  (4688-10356) | 6.3  (5.3-7.0) | 19.6  (5.5-42.4) | 4.4  (3.3-5.8) | 11.4 ± 2.3 |
| Rural | 6.6  (6.2-6.9) | 8395  (5617-11959) | 6.3  (5.5-7.0) | 24.2  (7.4-54.4) | 4.6  (3.5-5.8) | 11.1 ± 2.2 |
| Marital status ^[N=3646/3160]^ |  |  |  |  |  |  |
| Married | 6.6  (6.1-6.9) | 8108  (5315-11076) | 6.4  (5.5-7.1) | 23.1  (7.2-47.0) | 4.5  (3.4-5.8) | 11.2 ± 2.3 |
| Not married | 6.5  (5.9-6.9) | 6646  (4018-9707) | 6.1  (5.0-6.8) | 15.8  (4.2-40.9) | 4.4  (3.1-5.6) | 11.6 ± 2.4 |
| Education ^[N=3600/3123]^ |  |  |  |  |  |  |
| High | 6.6  (6.1-6.9) | 8260  (5587- 11098) | 6.3  (5.5-7.0) | 27.4  (9.5-51.2) | 4.4  (3.4-5.7) | 11.3 ± 2.1 |
| Intermediate | 6.6  (6.0-6.9) | 7749  (4990- 11032) | 6.3  (5.4-7.0) | 19.6  (5.8-43.9) | 4.7  (3.5-6.0) | 11.2 ± 2.4 |
| Low | 6.5  (5.8-6.9) | 5865  (3352- 9230) | 6.1  (5.0-6.9) | 10.9  (2.1-30.0) | 4.3  (2.9-5.6) | 11.7 ± 2.5 |
| Wealth ^[N=3032/2641]^ |  |  |  |  |  |  |
| Highest | 6.6  (6.1-6.9) | 8811  (6039-11625) | 6.4  (5.5-7.1) | 28.2  (10.9-55.3) | 4.6  (3.5-5.9) | 11.1 ± 2.0 |
| High | 6.6  (6.3-6.9) | 7643  (5263-10640) | 6.4  (5.6-7.1) | 21.4  (6.9-49.8) | 4.5  (3.4-5.7) | 11.2 ± 2.2 |
| Intermediate | 6.6  (6.1-6.9) | 6556  (3930-9507) | 6.3  (5.1-7.1) | 14.2  (4.1-34.9) | 4.3  (3.1-5.7) | 11.6 ± 2.4 |
| Low | 6.6  (6.1-6.9) | 6511  (3999-9252) | 6.1  (5.1-6.8) | 14.1  (2.9-33.5) | 4.3  (3.1-5.4) | 11.7 ± 2.4 |
| Lowest | 6.5  (5.3-6.7) | 6467  (3899-10161) | 6.0  (5.0-6.8) | 13.0  (3.7-31.6) | 4.4  (3.1-6.1) | 11.7 ± 2.7 |

Sample sizes vary across models owing to missing data for education, marital status, wealth, and urbanicity. The number of participants included in each wear time and movement behaviour analysis is indicated by the table superscripts.

SD, standard deviation; IQR, interquartile range; MVPA, moderate-to-vigorous physical activity; LPA, light physical activity; SB, sedentary behaviour.

**Table A6. Mean or median (SD or IQR) wear time and 24-hour movement behaviours across temporal characteristics.**

| **Characteristics** | **Wear time**  **(hours/day)** | **Step count**  **(steps/day)** | **Sleep duration**  **(hours/night)** | **MVPA**  **(minutes/day)** | **LPA**  **(hours/day)** | **SB**  **(hours/day)** |
| --- | --- | --- | --- | --- | --- | --- |
| N | 3648 | 3161 | 3161 | 3161 | 3161 | 3161 |
| Overall | 23.8  (23.3-24.0) | 7658  (4892-10779) | 6.3  (5.3-7.0) | 20.6  (5.8-45.0) | 4.5  (3.3-5.8) | 11.3 ± 2.3 |
| Weekday | 23.9  (23.4-24.0) | 7642  (4852-10882) | 6.3  (5.3-7.0) | 19.3  (5.2-44.4) | 4.4  (3.3-5.8) | 11.3 ± 2.4 |
| Weekend | 24.0  (23.4-24.0) | 7456  (4642-10891) | 6.4  (5.2-7.3) | 17.5  (0.0-45.7) | 4.3  (3.1-5.7) | 11.2 ± 2.5 |
| *P*-value | 0.21 | **0.0027** | **<0.0001** | 0.066 | **<0.0001** | **<0.0001** |
| Time of day |  |  |  |  |  |  |
| 00:00-05:59 | 5.9  (5.6-6.0) | 51  (16-125) | 4.7  (3.9-5.2) | 0.0  (0.0-0.0) | 0.1  (0.0-0.2) | 0.9 ± 0.9 |
| 06:00-11:59 | 6.0  (6.0-6.0) | 2754  (1617-4128) | 0.9  (0.4-1.5) | 4.1  (0.0-13.6) | 1.5  (1.0-2.0) | 2.3 ± 0.8 |
| 12:00-17:59 | 6.0  (6.0-6.0) | 3387  (2108-4896) | 0.0  (0.0-0.0) | 9.8  (2.3-23.4) | 1.8  (1.3-2.4) | 3.7 ± 0.9 |
| 18:00-23:59 | 6.0  (5.9-6.0) | 1206  (742-1906) | 0.5  (0.1-1.0) | 0.0  (0.0-5.2) | 1.0  (0.6-1.4) | 4.3 ± 0.8 |
| *P*-value | **<0.0001** | **<0.0001** | **<0.0001** | **<0.0001** | **<0.0001** | **<0.0001** |

SD, standard deviation; IQR, interquartile range; MVPA, moderate-to-vigorous physical activity; LPA, light physical activity; SB, sedentary behaviour.

**Table A7. Marginal mean (95% CI) wear time and 24-hour movement behaviours across social, economic, and environmental characteristics, after applying the core Wave 10 cross-sectional weights to the restricted sample^a^.**

| **Characteristics** | **Wear time**  **(days)** | **Step count**  **(steps/day)** | **Sleep duration**  **(hours/night)** | **MVPA**  **(minutes/day)** | **LPA**  **(hours/day)** | **SB**  **(hours/day)** |
| --- | --- | --- | --- | --- | --- | --- |
| N | 3044 | 2650 | 2650 | 2650 | 2650 | 2650 |
| Sex |  |  |  |  |  |  |
| Male | 5.8  (5.6-5.9) | 7930  (7620-8240) | 5.9  (5.7-6.0) | 34.4  (32.0-36.9) | 4.1  (4.0-4.3) | 11.9  (11.7-12.0) |
| Female | 5.6  (5.5-5.8) | 7400  (7149-7651) | 6.0  (5.9-6.1) | 22.7  (21.0-24.3) | 4.8  (4.7-4.9) | 11.1  (11.0-11.3) |
| Age (years) |  |  |  |  |  |  |
| 50-59 | 5.4  (5.2-5.7) | 9731  (9242-10220) | 6.3  (6.1-6.5) | 37.5  (33.7-41.2) | 5.2  (5.0-5.5) | 10.5  (10.3-10.8) |
| 60-69 | 5.7  (5.5-5.8) | 9043  (8643-9443) | 6.2  (6.1-6.3) | 38.2  (35.1-41.3) | 4.7  (4.6-4.9) | 11.1  (11.0-11.3) |
| 70-79 | 5.8  (5.7-6.0) | 7106  (6812-7401) | 6.0  (5.9-6.1) | 25.6  (23.5-27.7) | 4.3  (4.2-4.4) | 11.7  (11.5-11.8) |
| 80- | 5.9  (5.7-6.1) | 4781  (4421-5140) | 5.3  (5.1-5.5) | 12.9  (10.8-15.0) | 3.6  (3.4-3.8) | 12.7  (12.4-12.9) |
| Education ^[N=3013/2626]^ |  |  |  |  |  |  |
| High | 5.7  (5.5-5.8) | 8097  (7804-8390) | 6.1  (6.0-6.2) | 32.2  (30.0-34.4) | 4.5  (4.3-4.6) | 11.5  (11.3-11.6) |
| Intermediate | 5.8  (5.6-5.9) | 7862  (7486-8238) | 6.0  (5.9-6.1) | 28.6  (25.7-31.5) | 4.6  (4.4-4.8) | 11.4  (11.2-11.6) |
| Low | 5.6  (5.4-5.8) | 6854  (6445-7262) | 5.7  (5.5-5.9) | 23.6  (20.9-26.4) | 4.3  (4.1-4.5) | 11.7  (11.5-11.9) |
| Marital status ^[N=3011/2625]^ |  |  |  |  |  |  |
| Married | 5.8  (5.6-5.9) | 7890  (7617-8163) | 6.0  (5.9-6.1) | 29.4  (27.4-31.4) | 4.6  (4.4-4.7) | 11.3  (11.2-11.5) |
| Not married | 5.6  (5.4-5.8) | 7123  (6793-7453) | 5.8  (5.6-5.9) | 26.0  (23.7-28.4) | 4.3  (4.1-4.5) | 11.8  (11.6-12.0) |
| Wealth ^[N=2978/2595]^ |  |  |  |  |  |  |
| Highest | 5.6  (5.4-5.8) | 8519  (8050-8988) | 6.1  (5.9-6.2) | 35.3  (31.8-38.8) | 4.6  (4.4-4.8) | 11.2  (11.0-11.4) |
| High | 5.9  (5.7-6.1) | 7988  (7589-8386) | 6.1  (6.0-6.2) | 32.3  (28.9-35.7) | 4.4  (4.3-4.6) | 11.4  (11.2-11.6) |
| Intermediate | 5.8  (5.6-6.0) | 7092  (6686-7498) | 5.9  (5.8-6.1) | 25.7  (22.4-29.0) | 4.4  (4.2-4.6) | 11.7  (11.4-11.9) |
| Low | 5.7  (5.5-6.0) | 6868  (6346-7389) | 5.7  (5.6-5.9) | 24.0  (20.3-27.6) | 4.2  (4.0-4.4) | 11.9  (11.6-12.1) |
| Lowest | 5.2  (4.9-5.6) | 7112  (6516-7707) | 5.7  (5.4-5.9) | 21.4  (17.5-25.2) | 4.5  (4.2-4.8) | 11.7  (11.4-12.1) |
| Urbanicity ^[N=2978/2595]^ |  |  |  |  |  |  |
| Urban | 5.6  (5.5-5.7) | 7322  (7089-7555) | 5.9  (5.8-6.0) | 264  (24.8-28.0) | 4.4  (4.3-4.5) | 11.6  (11.4-11.7) |
| Rural | 5.8  (5.6-6.0) | 8168  (7722-8613) | 6.0  (5.8-6.1) | 32.2  (28.5-35.8) | 4.5  (4.3-4.7) | 11.5  (11.3-11.7) |

^a^ Core Wave 10 participants with valid accelerometry data.

Sample sizes vary across models owing to missing data for education, marital status, wealth, and urbanicity. The number of participants included in each wear time and movement behaviour analysis is indicated by the table superscripts.

The model for sex was adjusted for season of accelerometer wear and age group.

The model for age group was adjusted for season of accelerometer wear and sex.

The model for education was adjusted for season of accelerometer wear, age group, sex, and education.

The model for wealth was adjusted for season of accelerometer wear, age group, sex, education, and marital status.

The model for urbanicity was adjusted for season of accelerometer wear, age group, sex, education, marital status, and wealth.

95% CI, 95% confidence interval; MVPA, moderate-to-vigorous physical activity; LPA, light physical activity; SB, sedentary behaviour.

**Figure A1. Percentage of individuals who agreed to wear an accelerometer across social, economic, and environmental characteristics (N = 5,382).**

The horizontal lines show 95% confidence intervals.

**Figure A2. Marginal mean (95% CI) wear time across social, economic, and environmental characteristics (N = 3,648).**

The model for sex was adjusted for season of accelerometer wear and age group.

The model for age group was adjusted for season of accelerometer wear and sex.

The model for education was adjusted for season of accelerometer wear, age group, sex, and education.

The model for wealth was adjusted for season of accelerometer wear, age group, sex, education, and marital status.

The model for urbanicity was adjusted for season of accelerometer wear, age group, sex, education, marital status, and wealth.

95% CI, 95% confidence interval.

**Figure A3. Marginal mean (95% CI) time spent in moderate-to-vigorous physical activity across social, economic, and environmental characteristics (N = 3,161).**

The model for sex was adjusted for season of accelerometer wear and age group.

The model for age group was adjusted for season of accelerometer wear and sex.

The model for education was adjusted for season of accelerometer wear, age group, sex, and education.

The model for wealth was adjusted for season of accelerometer wear, age group, sex, education, and marital status.

The model for urbanicity was adjusted for season of accelerometer wear, age group, sex, education, marital status, and wealth.

MVPA, moderate-to-vigorous physical activity; 95% CI, 95% confidence interval.

**Figure A4. Marginal mean (95% CI) time spent in light physical activity across social, economic, and environmental characteristics (N = 3,161).**

The model for sex was adjusted for season of accelerometer wear and age group.

The model for age group was adjusted for season of accelerometer wear and sex.

The model for education was adjusted for season of accelerometer wear, age group, sex, and education.

The model for wealth was adjusted for season of accelerometer wear, age group, sex, education, and marital status.

The model for urbanicity was adjusted for season of accelerometer wear, age group, sex, education, marital status, and wealth.

LPA, light physical activity; 95% CI, 95% confidence interval.

**Figure A5. Marginal mean (95% CI) total sedentary time across social, economic, and environmental characteristics (N = 3,161).**

The model for sex was adjusted for season of accelerometer wear and age group.

The model for age group was adjusted for season of accelerometer wear and sex.

The model for education was adjusted for season of accelerometer wear, age group, sex, and education.

The model for wealth was adjusted for season of accelerometer wear, age group, sex, education, and marital status.

The model for urbanicity was adjusted for season of accelerometer wear, age group, sex, education, marital status, and wealth.

95% CI, 95% confidence interval.

**Figure A6. 24-hour profile of wear time by age group (N = 3,648).**

Means were adjusted for sex and ribbon represents the 95% confidence interval.

**Figure A7. 24-hour profile of step count by age group (N = 3,161).**

Means were adjusted for sex and ribbon represents the 95% confidence interval.

**Figure A8. 24-hour profile of sleep duration by age group (N = 3,161).**

Means were adjusted for sex and ribbon represents the 95% confidence interval.

**Figure A9. 24-hour profile of moderate-to-vigorous physical activity by age group (N = 3,161).**

Means were adjusted for sex and ribbon represents the 95% confidence interval.

MVPA, moderate-to-vigorous physical activity.

**Figure A10. 24-hour profile of light physical activity by age group (N = 3,161).**

Means were adjusted for sex and ribbon represents the 95% confidence interval.

LPA, light physical activity.

**Figure A11. 24-hour profile of total sedentary time by age group (N = 3,161).**

Means were adjusted for sex and ribbon represents the 95% confidence interval.
